# Leukocyte Telomere Length in the Neonatal Offspring of Mothers with Gestational and Pre-Gestational Diabetes

**DOI:** 10.1371/journal.pone.0163824

**Published:** 2016-10-13

**Authors:** Christopher Gilfillan, Pratyusha Naidu, Florence Gunawan, Fadwa Hassan, Pei Tian, Ngaire Elwood

**Affiliations:** 1 Eastern Health Clinical School, Monash University, Box Hill, Victoria, Australia; 2 Peninsula Health, Frankston, Victoria, Australia; 3 Cord Blood Stem Cell Research, Murdoch Children’s Research Institute, Parkville, Victoria, Australia; University of Newcastle, UNITED KINGDOM

## Abstract

**Aims:**

Telomeres undergo shortening with cell division, accelerated by increased oxidative stress. We aimed to demonstrate shortened telomeres in the offspring of mothers who have diabetes as a consequence of exposure to increased oxidative stress during intrauterine development.

**Methods:**

We examined the level of glycaemia (glucose, HbA1c, fructosamine), oxidative stress (lipid peroxidation) and the levels of antioxidant enzymes (Superoxide dismutase (SOD) and Selenium dependent glutathione peroxidase) and correlate these findings with mean telomere length (TL) in maternal and foetal blood in groups of pregnant women with pre-gestational diabetes (PGD), gestational diabetes (GD) and a euglycaemic control group.

**Results:**

Foetal and maternal glucose, maternal HbA1c, and foetal insulin and C-peptide were higher in the PGD group with the GD group being intermediate. Markers of oxidative stress did not vary between groups with the exception of foetal SOD activity that was highest in the GD group. There were no detectable differences in maternal or foetal TL between study groups. An exploratory analysis looking at correlations between glycaemic and oxidative stress parameters and TL revealed a negative correlation between maternal and foetal glucose and TL across the whole study population. This relationship held for the short-term marker of glycaemic control, fructosamine.

**Conclusions:**

We were unable to show significant telomere shortening in the offspring of mothers with PGD or GD. Exploratory analysis revealed a relationship between foetal TL and short-term glycaemia particularly in PGD. It is possible that increased telomerase activity can compensate for long-term increased oxidative stress but not for short-term dysglycaemia.

## Introduction

The view that an adverse intrauterine environment during pregnancy contributes to poor long-term health in the offspring is well established. There is some evidence that the offspring of women with pre-gestational type 1 diabetes, the archetypal adverse intrauterine environment, are at risk of developing glucose intolerance and cardiovascular disease in young childhood and adolescence [1].

Telomeres are nucleoprotein structures, located at the ends of chromosomes and are subject to shortening at each cycle of cell division. Telomeres consist of stretches of repetitive DNA with a high G-C content and are reported to be highly sensitive to damage induced by oxidative stress.[2] Telomere length shortens progressively during each round of cell division, accelerated by inflammation and oxidative stress, to a critical length, called the Hayflick limit, beyond which replicative senescence will be triggered. A number of associated proteins participate in the control of telomere length and include TRF1, TRF2, Ku86 and the enzyme telomerase. Telomerase itself consists of two components: the catalytic protein, a reverse transcriptase, (TERT), and the RNA template, (Terc)[3].

In humans, telomere length declines significantly with age and shorter leucocyte telomere length has been associated with T2DM [4]and its related condition such as obesity [5], insulin resistance [6], IGT [7]and atherosclerosis [8]. Despite these associations it remains unclear whether telomere shortening is a cause or a consequence of diabetes. Two recent studies have helped to clarify this. One study found that a shortened telomere length predicted the onset of diabetes in a high risk population independent of other risk factors [9]and in another prospective study revealed an adjusted Hazard Ratio of 2.00 for diabetes comparing the shortest verses the longest telomere length [10].

It is of interest to know whether the offspring of mothers who have diabetes (type 1, 2 or gestational diabetes) have shortened telomeres at birth as a consequence of exposure to increased oxidative stress during the many replicative cycles during embryogenesis in utero. A reduced telomere length in peripheral white blood cells has been associated with the presence of atherosclerosis in adults and if present in offspring of mothers with diabetes it could be another mechanism by which such offspring may carry an increased burden of disease in later life.

T2DM has been shown to be associated with elevated levels of oxidative DNA damage and decreased efficacy of DNA repair [11]. The extent of oxidative stress in mothers with diabetes and their offspring can be measured by the extent of lipid peroxidation and the activity of antioxidant enzymes. Assessment of malondialdehyde (MDA) has been used as the marker of lipid peroxidation. Among the different analytical methods established, the reaction with TBA (2-thiobarbituric acid) is the most widely used [12]. Diabetes is associated with increased oxidative stress and the level of TBARs in peripheral blood is elevated in diabetes [13]. TBARs are also elevated in mothers with diabetes and in the cord blood of their offspring [14]. Anti-oxidant enzyme activities (Glutathione-S-transferase (GST), selenium-dependent glutathione peroxidase (Se-GPx), catalase (CAT), and superoxide dismutase (SOD), and glutathione) also reflect oxidative stress [14].

In this study we will examine the level of glycemia (HbA1c, fructosamine), oxidative stress (lipid peroxidation by thiobarbituric acid reactive substances (TBARs)) and the levels of antioxidant enzymes (Superoxide dismutase (SOD) and Selenium dependent glutathione peroxidase (Se-GPx)) and correlate these findings with telomere length in peripheral blood leukocytes in maternal and foetal (cord) blood in groups of pregnant women with pre-gestational diabetes (n = 14, Type 1 n = 7, type 2 n = 7), gestational diabetes (n = 20) and a euglycaemic control group (n = 18). Our primary outcome measure, pre-specified, was a difference in mean telomere length between sub-groups by analysis of variance.

## Methods

### Recruitment and study population

The protocol was approved by the Peninsula Health Human Research and Ethics committee. All recruited patients were consented using approved patient information and consent forms and signed forms were co-signed by the investigator and kept in the patient’s file. Women were recruited from the population of women with gestational diabetes and pre-gestational (type 1 and type 2) diabetes attending a diabetes-in-pregnancy clinic at a single centre. Normal women were recruited from the antenatal clinic at the same hospital. Women with diabetes were managed according to the guidelines of the Australia Diabetes in Pregnancy Society. Gestational diabetes was diagnosed on the basis of a 50g glucose challenge test at 26 weeks and if positive (1 hour blood sugar level greater that 8.0mmol/L) a follow up 75 g glucose tolerance test was performed at 28 weeks. Gestational diabetes was diagnosed if the fasting blood glucose was greater than 5.4 mmol/L or the 2-hour value exceeded 8.0 mmol/L. If the capillary glucose levels exceeded 5.4 mmol/L fasting or 7.0mmol/L post-prandially despite adherence to the dietary and exercise prescription insulin therapy was commenced and adjusted to achieve those thresholds. The dietary prescription involved the use of low glycaemic index carbohydrates spread evenly through the day and restriction of dietary fat. Patients were encouraged to remain physically active but no formal exercise program was offered. For obese patients a goal of weight maintenance during pregnancy was established. For those that required insulin a variety of regimens was used from once daily basal insulin to four times daily basal-bolus regimens to insulin pump devices.

### Sample handling

Maternal peripheral blood and cord blood were collected at the time of delivery in heparinized tubes. Aliquots were taken for HbA1c, fructosamine, glucose, c-peptide and insulin determinations. After centrifugation buffy coat and plasma were removed and stored at -80°C for assays of telomere length and TBARs respectively. Red cells were washed with normal saline and then lysed in chilled distilled water; the lysate is diluted in phosphate buffer and stored at -80°C for assay of enzyme activities.

### The measurement of oxidative stress in maternal and cord blood

Malondialdehyde (MDA) is a naturally occurring product of lipid peroxidation. Lipid peroxidation is a well-established mechanism of cellular injury in both plants and animals and is used as an indicator of oxidative stress in cells and tissues. Lipid peroxides derived from polyunsaturated fatty acids are unstable and decompose to form a complex series of compounds, which include MDA.

The measurement of Thiobarbituric Acid Reactive Substances (TBARS) is a method for screening and monitoring lipid peroxidation. We used Cayman’s TBARS assay kit (Cat no 10009055) to assay lipid peroxidation in plasma samples. The MDA-TBA adducts formed by the reaction of MDA and TBA under high temperature (90–100°C) and acidic conditions is measured colorimetrically at 530–540nm. Typically normal human serum or plasma has a lipid peroxide level (expressed in terms of MDA) of 1.86–3.94μM.

Superoxide dismutase (SOD) is an enzyme that catalyses the dismutation of the toxic superoxide radical to hydrogen peroxide and oxygen. We measure SOD activity using a commercial kit (Ransod cat no SD 125, Randox Laboratories, UK). Briefly this assay uses the reaction of xanthine with xanthine oxidase to produce superoxide radicals which in turn react to form a red formazan dye. The degree of inhibition of this reaction correlates with the SOD activity in the sample under assay conditions.

Selenium-dependent glutathione peroxidise (Se–GPX) activity is measured by the method of Paglia and Valentine using a commercial kit (Ransel Cat No RS 504 Randox laboratories, UK). Glutathione Peroxidase catalyses the oxidation of Glutathione by Cumene Hydroperoxide. In the presence of Glutathione Reductase and NADPH the oxidised Glutathione is immediately converted to the reduced form with a concomitant oxidation of NADPH to NADP+·. The decrease in absorbance at 340 nm is measured.

### Measurement of telomere length

#### Genomic DNA isolation and dilution

Genomic DNA (gDNA) was extracted using GenElute^TM^ Mammalian Genomic DNA Miniprep Kit (Sigma, Cat G1N70) according to the instructions of manufacturer for whole blood preparation. The concentration and quality of the gDNA obtained was determined by NanoDrop spectrophotometer (Biolabs) and agarose gel electrophoresis. The gDNA samples were diluted in 1xTE buffer (10mM Tris-HCI, 1mM EDTA, pH 8.0) to a final concentration of 4 ng/μl).

#### Absolute quantitative real-time PCR assay

Average telomere length was measured from gDNA by using a quantitative real-time PCR (qRT-PCR) method previously described [15] with the following modifications.

A standard curve was generated by performing serial dilutions 1 in 3 (6 dilutions). IKb plus DNA ladder (Invitrogen Cat. No. 10787–018) was added to each standard dilution to maintain a constant final concentration of DNA of 4ng/ μl.

Instead of setting up separate oligomer standard curves for TTAGGG repeats and 36B4 (the control for genome copy number), to reduce PCR variation between each run, a standard curve was established by mixing TTAGGG repeats (PT9S) and 36B4 (PT8S) oligonucleotides at dilutions to ensure that test DNA cycle threshold values were within the linear range of the two standard curves.

To reduce the variation introduced by pipetting, the pair of forward and reverse primers were mixed together, aliquoted into an appropriate volume for each run and then stored at -20°C.

All PCR reactions were prepared using QIAgility (Qiagen) and reactions were run on a RotorGene 6000 (Corbett). Each sample was analysed in triplicate. Thermal cycler reaction conditions were set at 50°C for 2 min, 95°C for 5 min followed 30 cycles of 95°C for 15 s, 56°C for 60 s (data was acquired from the Green channel at this step), ending with a melt step. Reactions were set up for 36B4 in the same way as that for the telomere primers except only 150 nM per reaction for forward primer (5’ CAGCAAGTGGGAAGGTGTAATCC 3’) and reverse primer (5’ CCCATTCTATCATCAACGGGTACAA 3’) was used; reaction conditions were set at 50°C for 2 min, 95°C for 5 min followed 35 cycles of 95°C for 15 s, 60°C for 60 s (data was acquired from the Green channel at this step), ending with a melt step.

Real-time PCR data was analysed using the Rotor-Gene 6000 software. The calculated amount of template was exported into MS Excel where the final telomere length was determined according to O'Callaghan, N [15]

#### Terminal restriction fragments (TRF) assay

Standard TRF assay was performed to verify telomere length obtained using absolute qRT-PCR telomere method. TRF Assay was performed using *T*elo*TAGGG* Telomere Length Assay (Roche Cat 12 209 136 001) according to the instructions of manufacturer.

### Statistical methods

The effect of diabetes category was assessed for each parameter using analysis of variance and where the parameter was not normally distributed the Kruskal-Wallis comparison of medians was used. Where appropriate, Student’s t-test was used to compare individual categories. Relationships between glycaemic parameters and measurements of oxidative stress with foetal telomere length were explored using Pearson’s correlation co-efficient.

## Results

There were a total of 52 subjects with singleton pregnancies resulting in 52 live births. Twenty subjects had gestational diabetes. 14 patients had pre-gestational diabetes 7 of these were type 1 and the remainder had type 2 diabetes. The mean maternal BMI was in the obese range but there were no statistical differences between groups (Table 1). Application of treatment guidelines (see above) resulted in all pre-gestational subjects with diabetes and 18 of the 20 gestational diabetes patients being treated with insulin. In the gestational diabetes subjects insulin doses ranged from 4 to 52 units daily.

**Table 1:** Maternal and foetal characteristics in women with Gestational Diabetes, Pre-gestational Diabetes and controls.

	ALL	Control	GDM	PGDM	ANOVA
**Number**	52	18	20	14	
**Maternal BMI (kg/m**^**2**^**)**	29.4 ± 8.9	28.2 ± 7.1	30.4 ± 10.4	29.5 ± 7.1	NS
**Insulin use (percent, mean dose (U))**			90%, 13.5	100%, 13.5	NS
**Male: Female offspring**	25:27	8:10	12:8	5:9	NS
**Birthweight (g)**	3854 ±74	3829 ±116	3864 ±6	3880 ±4	NS

### Foetal and Maternal Glycaemic parameters

The average maternal HbA1c was 5.63% (38mmol/mol) with a significant trend to a higher HbA1c in the pre-gestational diabetes group, where the value reached the diabetic range (>6.0% (42 mmol/mol)). The HbA1c was well-controlled during pregnancy in both groups with diabetes. Plasma glucose at the time of delivery was higher in the group with pre-gestational diabetes (p<0.01) and this was reflected in higher cord blood glucose in offspring (p<0.01). Foetal insulin levels were also higher in the pre-gestational diabetes group. Although the ANOVAs showed a significant effect of diabetes category this was driven by the pre-gestational group with the gestational diabetes group not differing from controls with respect to any of these parameters. Foetal C-peptide reflected the changes in foetal insulin (Table 2). Maternal and foetal fructosamine was not different between groups (data not shown) and interpretation of maternal insulin and C-peptide levels were compromised by the presence of subjects with type 1 diabetes in the pre-gestational diabetes group (data not shown).

**Table 2:** Glycaemic parameters in maternal plasma and foetal (umbilical cord) plasma at birth in control and diabetic women.

	ALL	Control	GDM	PGDM	ANOVA
**Number**	52	18	20	14	
**Maternal HbA1c % (mmol/mol)**	5.6 ± 0.7 (38 ± 5)	5.4 ± 0.3 (36 ± 2)	5.6 ± 0.6 (38 ± 5)	6.1 ± 0.9 (43 ± 6)	p<0.01
**Maternal Glucose (mmol/L)**	6.7 ± 3.6	5.6 ± 1.2	5.8 ± 2.0	9.4 ± 5.8	p<0.01
**Foetal Glucose (mmol/L)**	4.6 ± 1.7	3.8 ± 1.0	4.5 ± 1.4	5.6 ± 2.4	p<0.01
**Foetal Insulin (mU/L)**	7.0 ± 11.8	2.4 ± 3.2	4.2 ± 6.0	17.1 ± 18.3	p<0.005*
**Foetal C-Peptide (nmol/L)**	0.38 ± 0.28	0.27 ± 0.12	0.36 ±0.20	0.53 ± 0.44	p<0.05*

*Not normally distributed so analysed by Kruskal-Wallis comparison of medians.

### Markers of oxidative stress and Telomere length

Although there was a trend to higher TBARS in mothers and offspring with pre-gestational diabetes, there were no statistical differences between TBARS measurements in maternal or foetal plasma in any group (Table 3). The anti-oxidant enzyme activities showed a trend to be higher in the GDM group in both foetal and maternal plasma. This reached significance for foetal SOD where levels in offspring of women with gestational diabetes were higher than both control and mothers with pre-gestational diabetes (p<0.05). Telomere length was significantly longer in foetal compared to maternal peripheral white blood cells. There was no difference in telomere length between groups in either maternal or foetal peripheral white blood cells (Table 3). Thus the primary endpoint was not met.

**Table 3:** Foetal and maternal markers of oxidative stress and telomere length in gestational and pre-gestational diabetic pregnancies and controls.

	ALL	Control	GDM	PGDM	ANOVA
**Maternal TBARs (μM)**	23 ± 10	22 ± 10	22 ± 8	28 ± 12	NS
**Foetal TBARs (μM)**	24 ± 10	22 ± 8	25 ± 11	27 ± 12	NS
**Maternal SOD**	288 ± 99	258 ± 53	333 ± 136	264 ± 60	NS
**Foetal SOD**	298 ± 105	262 ± 64	346 ± 133	270 ± 73	p < 0.05
**Maternal Se-GPx**	6271 ± 1990	6069 ± 1169	6579 ± 2542	6057 ± 1812	NS
**Foetal Se-GPx**	4226 ± 1387	4085 ±1128	4511 ± 1843	4043 ± 997	NS
**Maternal telomere length (kb)**	7.2 ± 1.3	7.0 ± 1.3	7.2 ± 1.4	7.5 ± 1.2	NS
**Foetal telomere length (kb)**	10.6 ± 1.9	10.4 ± 1.6	10.9 ± 1.6	10.7 ± 2.4	NS

### Relationships between glycaemic parameters oxidative stress and foetal telomere length

**Fig 1:**
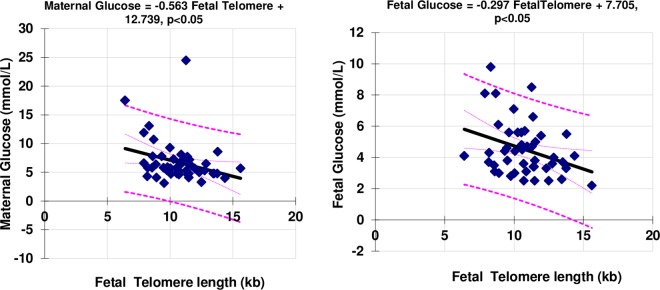
The relationship between maternal and foetal glucose at delivery (mmol/L) and foetal telomere length (kb).

An exploratory analysis of the relationships between foetal telomere length and indicators of glycaemic control and oxidative stress within the categories of diabetes within the study was performed. There was a negative correlation between maternal glucose and foetal glucose with foetal telomere length across the whole study population (Fig 1). Further analysis showed that this was due to a striking relationship between maternal glucose and foetal telomere length in the subjects with pre-gestational diabetes, with higher glucose leading to shorter telomere length (p<0.001). This relationship held for the short-term marker of glycaemic control, fructosamine, but not for the longer-term marker haemoglobin A1c. Within this group (pre-gestational diabetes), higher maternal glucose was associated with higher maternal TBARS, which in turn predicted a smaller telomere length (p<0.001). Even in this sub-group analysis there were no relationship between glycaemic parameters, foetal or maternal telomere length with the anti-oxidant enzyme activities, SOD and Se-GPx.

The relationship of acute glycaemia and foetal telomere length did not reach significance in the gestational diabetes group or the control group, nor did separate examination of those gestational diabetes patients who used insulin increase the strength of the relationship.

## Discussion

Our primary analysis was unable to show significant telomere shortening in the offspring of mothers with pre-gestational or gestational diabetes. This is consistent with finding of Cross *et*. *al*. who were unable to find telomere shortening in the young adult offspring of mothers with diabetes [16]. However, a recent study did show a significant shortening in telomere length of foetal leucocytes in offspring of mothers with GDM compared to normal pregnancy [17]. Cross *et*. *al*. were also unable to find a relationship between maternal diabetes and cord blood telomere length although they did find increase telomerase activity in cord blood from gestational and type one mothers with diabetes [18]. This can be interpreted as a response to the added stress on telomeres in these offspring. Other authors have found telomere shortening in obese children [19] and in offspring of mothers exposed to neuropsychological stress during pregnancy [20]. Telomere shortening has been observed in trophoblast from placental tissue of mothers with poorly controlled diabetes but not in cord blood leukocytes in the same study [21].

It should be noted that our patients were very well controlled and aggressively treated with insulin. This may have limited our ability to see differences between groups. Insulin therapy will lead to only small differences in glycaemic parameters and will ameliorate the oxidative environment. Telomerase activity may be induced by insulin [22] also limiting our ability to discern differences in telomere length between groups.

Many studies have demonstrated that telomere length is an inheritable trait [23] with both maternal and paternal contributions [24]. There is also a contribution from paternal age [25]. We do not have information on paternity of our offspring. Foetal and maternal telomere lengths were not correlated in our study but we cannot exclude genetic influences on foetal telomere length as confounding factors.

What we have shown in a secondary exploratory analysis is that foetal hyperglycemia (and hyperinsulinemia) found at delivery, particularly in our population with pre-gestational diabetes, was associated with elevated markers of lipid peroxidation and shortened telomeres. This relationship also held with the short-term marker of glycaemic control, fructosamine. Fructosamine is the name given to glycated lysines within serum proteins that have a reducing capacity that can be detected in a colorimetric reaction [26]. The principle serum protein is albumin and as albumin has a half-life of 14 days the measured fructosamine reflects glycaemic control over that time. In pregnancy fructosamine falls because of changing albumin dynamics and dilution anemia and these factors may contribute to differences across our study groups. Nevertheless our findings suggest that glycemia with the past fortnight may be a relevant determinant of foetal telomere length. It is possible that this relationship reveals oxidative stress acting to shorten telomeres in the medium term with the action of induced telomerase activity repairing this damage over a longer time frame. Telomere length is a dynamic parameter that is capable of short-term change [27] and this may be why we can only find relationships with short–term markers of glycaemic control, while longer-term perturbations are corrected by increased telomerase activity which in turn may be promoted by the action of insulin at least in rats [28]. We have not measure telomerase activity in the current study and so cannot confirm the observation of Cross *et*. *al*. [18] that telomerase activity is elevated in offspring of mothers with diabetes.

Although telomere shortening may be a consequence of increased oxidative stress in patients with diabetes, there is emerging evidence that telomere shortening may predispose individuals to type 2 diabetes with genetic polymorphisms in telomere pathway genes linked to an increased incidence of type 2 diabetes[29]. Shortened telomeres have also been linked to increased beta–cell senescence and failure [30]. Shortened telomeres are also a feature of diabetic complications, particularly atherosclerosis. Prospective studies have now shown that leukocyte telomere length is independently associated with the risk of incident diabetes in both high-risk and moderate-risk populations [9, 10]. Although we were unable to demonstrate a reduction in telomere length in the offspring of mothers with diabetes we were able to show some evidence of glucose-related telomere shortening and this may still be a mechanism whereby the intra-uterine environment predisposes such offspring to diabetes and atherosclerotic disease later in life.

Alternatively, the currently available evidence suggests that glucose-related telomere shortening at birth is a transient phenomenon and does not persist into adult life and may not have a lasting influence on the risk of type 2 diabetes and metabolic complications in offspring.

## Supporting Information

S1 Table Individual patient data for all participants and their offspring. (XLSX)
